# Scalar Fluxes Near a Tall Building in an Aligned Array of Rectangular Buildings

**DOI:** 10.1007/s10546-017-0308-4

**Published:** 2017-11-04

**Authors:** Vladimír Fuka, Zheng-Tong Xie, Ian P. Castro, Paul Hayden, Matteo Carpentieri, Alan G. Robins

**Affiliations:** 1 Aerodynamics and Flight Mechanics Group, University of Southampton, Southampton, SO17 1BJ UK; 2 EnFlo, University of Surrey, Guildford, GU2 7XH UK

**Keywords:** Atmospheric dispersion, Large-eddy simulation, Tall building

## Abstract

Scalar dispersion from ground-level sources in arrays of buildings is investigated using wind-tunnel measurements and large-eddy simulation (LES). An array of uniform-height buildings of equal dimensions and an array with an additional single tall building (wind tunnel) or a periodically repeated tall building (LES) are considered. The buildings in the array are aligned and form long streets. The sensitivity of the dispersion pattern to small changes in wind direction is demonstrated. Vertical scalar fluxes are decomposed into the advective and turbulent parts and the influences of wind direction and of the presence of the tall building on the scalar flux components are evaluated. In the uniform-height array turbulent scalar fluxes are dominant, whereas the tall building produces an increase of the magnitude of advective scalar fluxes that yields the largest component. The presence of the tall building causes either an increase or a decrease to the total vertical scalar flux depending on the position of the source with respect to the tall building. The results of the simulations can be used to develop parametrizations for street-canyon dispersion models and enhance their capabilities in areas with tall buildings.

## Introduction

Dispersion of atmospheric pollution or suddenly released hazardous materials in urban areas is widely regarded as an important issue influencing the health and safety of the population. It is important to be able to predict the area affected by the plume resulting from an accidental or deliberate release of a dangerous substance inside a city and the concentrations of the pollutant in the area. When dealing with accidental or deliberate releases of contaminants the sources are usually localized in space to a small area or volume where the release happens. The release may also be localized in time.

At large distances from a continuous release the scalar plume characteristics do not depend too much on the exact geometry of the streets in the given location or on the exact location of the source within the street network (Theurer et al. 1996; Belcher 2005). One can often successfully use Gaussian dispersion models for the prediction of scalar concentrations at a sufficient distance from the source (Davidson et al. 1995; Macdonald et al. 1998). In the near-field of the release the dispersion pattern can be strongly non-Gaussian and the exact locations of the source and/or the buildings near the concentration measurement location are important (Theurer et al. 1996; Xie and Castro 2009).

Many past experimental and computational studies concentrated on flow or scalar dispersion in an idealized urban roughness. The flow and turbulence in uniform arrays of cubes were examined experimentally in field and wind-tunnel experiments by, for example, Macdonald et al. (2002) and Inagaki and Kanda (2008, 2010). Dispersion through an array of elongated buildings was examined experimentally by Macdonald et al. (1998) and Yee et al. (2006), who focused on concentration fields and concentration variance in the far field where the plume has an approximately Gaussian distribution.

The numerical studies of Coceal et al. (2006, 2007) investigated flow and turbulence in an aligned and staggered array of cubes using direct numerical simulation. Garbero et al. (2010) simulated dispersion processes experimentally in a densely packed street network with uniform height, and Branford et al. (2011) examined scalar dispersion from localized sources in an aligned array of cubes for different wind directions. They identified six main processes that control the dispersion in the near-field of the source: advection or channelling in the streets, lateral dispersion due to turbulence and dividing streamlines, plume skewing due to the flow turning with height, detrainment by turbulent dispersion or mean recirculation, entrainment to building wakes and secondary sources and plume meandering.

The effect of non-uniform (“random”) heights of the buildings in the staggered building array on flow and turbulence was studied experimentally by Cheng and Castro (2002) and by Xie et al. (2008) using large-eddy simulation (LES). Xie et al. (2008) showed that relatively larger buildings in the array contribute disproportionately to the surface drag and that the local flow can be influenced by relatively remote blocks. Buildings in the wake of the taller building are shielded and contribute less to the total drag. Boppana et al. (2010) then investigated scalar fluxes in uniform height and random height arrays from a large scalar area source with constant concentration and found considerably more complex vertical scalar-flux patterns in the random height array including regions of counter-gradient turbulent flux.

Recently, Goulart et al. (2016) simulated continuous releases from localized sources in an aligned array of cubes, and mainly investigated the spatio-temporal variability of the mean concentration with two wind directions, and found considerable differences in the transport and diffusion mechanisms when the flow is parallel to the street direction and when it is oblique (at a $45^{\circ }$ angle). In the streets parallel to the flow direction the mixing was reduced and high spatial and temporal variability was observed.

In modern cities one can often find buildings that surmount the surrounding canopy. These tall buildings can be isolated or form a group, typically in modern city centres; isolated tall buildings are the topic of this paper. Heist et al. (2009) experimentally and numerically examined the flow around an isolated building in a regular neighbourhood of buildings forming streets and closed courtyards. They noted large flow speeds in the spanwise direction that were caused by the presence of the tall building and vertical velocities downwind of the tall building reaching 25% of the freestream velocity. Brixey et al. (2009) used the same building configuration as Heist et al. (2009) for wind-tunnel and numerical simulations of scalar dispersion from line sources. They found that the vertical dispersion and the vertical extent of the plume in the wake of the tall building are greatly enhanced, while the spanwise flow towards the tower also increased the width of plumes from sources further away from the tall building laterally.

In recent years it has become possible to measure temporally-resolved flow and scalar concentrations simultaneously in a wind tunnel (Carpentieri et al. 2012), enabling direct measurements of the turbulent scalar flux that may be an important contributor to the transport of pollutants from the street network to the flow above (Caton et al. 2003; Salizzoni et al. 2011). Scalar fluxes, including the turbulent scalar flux, in a three-dimensional street canyon and a street intersection were measured by Nosek et al. (2016, 2017) with a focus on the influence of roof height non-uniformities on pollution dispersion.

Project DIPLOS (Dispersion of Localised Releases in a Street Network)^1^ aims to increase our understanding of the dispersion processes in street networks for localized scalar sources by means of wind-tunnel experiments and computer simulations. The ultimate goal of the project is to develop new parametrizations for street-network dispersion models (e.g., Belcher 2005; Soulhac et al. 2013). This class of dispersion models considers discrete parts of the street network as control volumes with certain concentration values and computes concentration fluxes between these control volumes. To be able to derive parametrizations for scalar fluxes in street-network models it is necessary to know the value of scalar fluxes between individual streets, intersections, empty areas and the boundary layer above the canopy. It is also necessary to understand how they depend on factors such as the source position and wind direction with respect to the street orientations.

The idealized urban geometry chosen herein is similar to that of Branford et al. (2011). However, the arrays of cubes do not form long streets that are typical of European city centres and that are typically described by street-network dispersion models. The chosen geometry uses blocks with dimensions $h \times 2h \times h$ so that the streets in one direction are two times longer than their width and height. Earlier, Castro et al. (2017) concentrated on the flow and turbulence in the array of rectangular buildings by means of wind-tunnel experiments and numerical simulations, i.e. using LES and direct numerical simulation (DNS). One of the findings was the high sensitivity of the results to small uncertainties in the experimental set-up and the difficulty of measuring quantities in the same position relative to the buildings in different locations in the array. The simulations showed that a typical street-canyon flow develops in streets that are 2*h* long and therefore the chosen configuration is suitable for the purpose of street-network model parametrization. The simulated flow agreed well with the measurements inside the canopy region and above up to $z/h \approx 3$, where *z* is the vertical coordinate.

Here we present results of the corresponding scalar dispersion fields arising from a ground-level source within the urban canopy. In addition to the earlier set-up in the previous paper, the effect of a tall building in the array is also considered. The paper is organized as follows: experimental and numerical methods used for the wind-tunnel experiments and LES and the set-up of test cases are introduced in Sect. 2. The results of the simulations of the uniform building array are introduced in Sect. 3, while Sect. 4 shows the results of the same array with one building three times as tall as in the original set-up. Section 5 summarizes the conclusions.

## Methodologies

### Wind-Tunnel Experiments

The experiments were conducted in the EnFlo environmental wind tunnel at the University of Surrey. This is an open-circuit tunnel with a working section that is $20\,\mathrm {m}$ long and $3.5\,\mathrm {m}\times 1.5\,\mathrm {m}$ in cross-section. The model canopy comprised a square array of 294 ($14\times 21$—in *x* direction $\times y$ direction) rectangular blocks with $x \times y \times z$ dimensions $h \times 2h \times h$, where the height $h=70\,\mathrm {mm}$. The blocks were mounted on a turntable whose axis of rotation was $14\,\mathrm {m}$ downstream of the test-section entrance. The origin of the rectangular coordinate system was set at the turntable (and model) centre, with *x* in the streamwise direction and *z* upwards. A more detailed description of the uniform array model and the approach flow can be found in Castro et al. (2017). The 1-$\mathrm {m}$ deep simulated boundary layer was well within the fully-rough-wall regime.

**Fig. 1:**
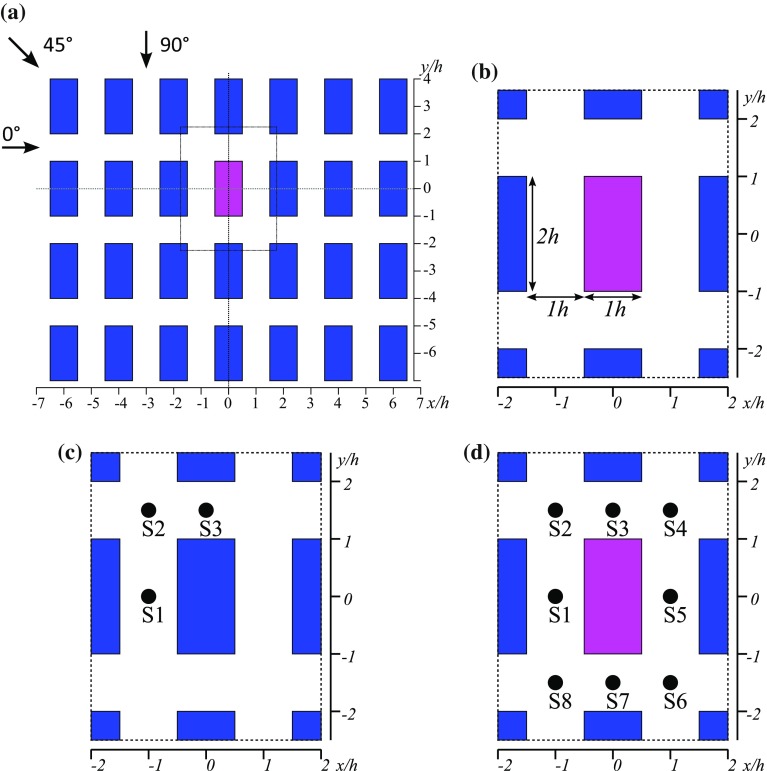
The layout of the test case. **a** A part of the experimental building array, the coordinate system and the wind directions. The magenta building is replaced by the tall building in the tall building scenario. **b** Dimensions of the buildings and streets. **c** Source position numbers for the uniform buildings case. **d** Source position numbers for the tall building case. The coordinate axes are fixed to the building array

Two reference ultrasonic anemometers mounted downstream of the array in the tunnel exit ducts were used to ensure that all the experiments were undertaken at the same freestream velocity in the approach flow ($2\,\mathrm {m}\,\mathrm {s}^{-1}$). Velocity and turbulence measurements were made using a two-component Dantec laser Doppler anemometer (LDA) system with a FibreFlow probe of outside diameter $27\,\mathrm {mm}$ and focal length $160\,\mathrm {mm}$. This provided a measuring volume with a diameter of $0.074\,\mathrm {mm}$ and a length of $1.57\,\mathrm {mm}$. Measurements in the local *x*–*z* plane within the street network (i.e. in planes aligned with the streets, where *z* is the vertical axis and *x* is along the short streets, see Fig. 1) were obtained by use of a small mirror set at $45{^\circ }$ beneath a downward pointing probe. The flow was seeded with micron-sized sugar particles at a sufficient level to attain data rates around $150\,\mathrm {Hz}$.

Tracer concentration measurements were performed by releasing a neutrally buoyant gas “tracer” into the flow and measuring its concentration using air sampling at selected points downstream. The tracer used was a gas mixture of propane in air and the emission was released from a round source with a $20\,\mathrm {mm}$ internal diameter that both removed sensitivity to the source position in the street and minimized effects of emission momentum. The results presented here refer to source location S1 in Fig. 1, which was located $70\,\mathrm {mm}$ upstream of the origin. The instrument used for concentration measurements was a Cambustion fast flame ionisation detector (FFID), a fast response instrument that is capable of measuring hydrocarbon concentration fluctuations with a frequency response of $200\,\mathrm {Hz}$. In general, data collection times were $2.5\,\mathrm {min}$, as described in Castro et al. (2017).

Scalar fluxes were measured using the LDA and FFID instruments at the same time on a common measurement volume. This set-up was described by Carpentieri et al. (2012) and is capable of measuring the turbulent part of the flux along with the mean part at locations within the urban model.

Data from two measurement campaigns are presented: the original set of data was already used in Castro et al. (2017) and is referred to as “wind tunnel 2015” (or shortened as “w.t. 2015”) in figures. Scalar fluxes were measured only in horizontal directions and only the uniform height array was used. A new set of data was collected in 2016, which includes vertical scalar fluxes and measurements in the array with a single tall building. It is denoted “wind tunnel 2016” or “w.t. 2016” in figures. The experiments are summarized in Table 1.

The measurements were conducted in a much larger number of locations in the array than those used for comparison herein. These results are available for further analysis.

### Large-eddy Simulation

In LES the fields of flow variables are spatially filtered, where the filtered Navier–Stokes equations are solved. We consider these equations with an eddy viscosity subgrid model

1 $$\begin{aligned} \frac{\partial \varvec{u}}{\partial t}+\nabla \cdot (\varvec{uu})&=-\frac{\nabla p}{\rho }+\nabla \cdot \left( \left( \nu +\nu _{\mathrm {sgs}}\right) \nabla \varvec{u}\right) , \end{aligned}$$

2 $$\begin{aligned} \nabla \cdot \varvec{u}&=0, \end{aligned}$$

where $\varvec{u}$ is the filtered (resolved) velocity field, *p* is the pressure field, $\nu$ is the molecular viscosity of the air and $\nu _{\mathrm {sgs}}$ is the subgrid-scale viscosity that has to be determined by a subgrid stress model. The Navier–Stokes equations are accompanied by the transport equation of a passive scalar,

3 $$\begin{aligned} \frac{\partial c}{\partial t}+\nabla \cdot (\varvec{u}c)&= \nabla \cdot \left( \left( \kappa +\kappa _{\mathrm {sgs}}\right) \nabla c\right) , \end{aligned}$$

where *c* is the scalar concentration, $\kappa$ is the scalar diffusivity and $\kappa _{\mathrm {sgs}}$ is the subgrid scalar diffusivity that has to be modelled. Here we assume that the scalar diffusivity can be computed as

4 $$\begin{aligned} \kappa _{{\mathrm {sgs}}}&= \frac{\nu _{\mathrm {sgs}}}{{Sc_{\mathrm {sgs}}}}, \end{aligned}$$

where ${{Sc_\mathrm{sgs}}}$ is the subgrid Schmidt number that can be defined as a constant in the subgrid model.

Two numerical codes for LES are used: the first is the open-source CFD package OpenFOAM version 2.1. The selected solver channelFoam is intended for simulations of incompressible flow with periodic boundary conditions. It maintains a constant flow-rate by adjusting the spatially-uniform pressure gradient that represents the volume force driving the flow. The solver uses the PISO method (Issa 1986) on a cell-centred grid. The mixed time-scale subgrid eddy viscosity model of Inagaki et al. (2005) was selected for the simulations because of the robustness and dynamic adaptation to local flow conditions demonstrated by Inagaki et al. (2005). No wall model is used in OpenFOAM in this study.

The second code used is the Extended Large Eddy Microscale Model (ELMM), an in-house CFD code developed from the Charles University Microscale Model (CLMM, see Fuka and Brechler 2011). ELMM was developed specifically for problems of flow and dispersion in complex geometry in the atmospheric boundary layer, and uses the projection method on a staggered orthogonal grid to solve the incompressible Navier–Stokes equations. For details about the numerical method see Fuka (2015). For the present simulations the subgrid model by Nicoud et al. (2011) was selected. The mixed-time scale model was not available in ELMM, but Nicoud et al. (2011) also demonstrated good robustness and adaptivity to the local flow. A wall model computing the wall shear stress from instantaneous velocities using a logarithmic law of the wall is enabled.

**Table 1:** Configurations of the wind-tunnel experiments

Campaign	Buildings	Wind dir. ($^{\circ }$)	Source positions
2015	Uniform	0	S1, S2, S3
2015	Uniform	45	S1, S2, S3
2015	Uniform	90	S1, S2, S3
2016	Uniform	0	S1, S2, S3
2016	Uniform	45	S1, S2, S3
2016	Uniform	90	S1, S2, S3
2016	Tall building	0	S2

Only horizontal scalar fluxes were measured in the 2015 measurement campaign. More data are available in the accompanying data repository

Both subgrid models used return zero eddy viscosity for laminar sheared flows and do not require any wall-damping functions. ELMM was used only for a limited set of computations to confirm the accuracy of the OpenFOAM results and to test the sensitivity to small changes in the wind direction. The presented LES results were produced with OpenFOAM unless stated otherwise.

### Adopted Set-up

#### Uniform Height Array

The most commonly studied type of regular obstacle array, in the context of atmospheric dispersion in urban areas (e.g., (Coceal et al. 2006, 2007; Branford et al. 2011)), is the regular array of cubes in non-staggered or staggered layouts. The streets in arrays of cubes are not the best approximation for the streets in European cities, which commonly form street canyons that are considerably longer than the width of the street. Castro et al. (2017) show that the chosen arrangement has streets “just long enough to be representative for the street network modelling approach”.

The basic obstacle layout studied is the regular array of identical building blocks with dimensions $1\,h$(length)$\times 2\,h$(width)$\times 1\,h$(height) with buildings laid out in an orthogonal array in which all streets are $1\,h$ wide. The layout is depicted in Fig. 1. Within a repeating unit streets parallel to the *x* axis are $1\,h$ long and will be called “short streets” hereafter, streets parallel to the *y* axis are $2\,h$ long and will be called “long streets”.

#### Tall Building in the Regular Array

In addition to the building array with uniform height we introduced one taller building with height $3\,h$ with the same horizontal dimensions as the base building. The height of the building was chosen to be small enough to ensure that it remained within the turbulent boundary layer that was generated in the wind tunnel by all the other obstacles (in the absence of the tall building). The tall building replaced one of the buildings in the array. Due to the periodic boundary conditions the tall building is not completely isolated, and is assumed to be a part of a larger periodic array where the streamwise distance between tall buildings is $8\times 3\,h$ and the lateral distance is $4\times 3\,h$.

#### LES Set-up

All computations used a uniform Cartesian grid with resolution $\Delta =h/16$. The base domain used for the uniform array of buildings had dimensions $12\,h$ (length) $\times 12\,h$ (width) $\times 12\,h$ (height). For the tall building, the domain was extended two times in the *x* direction, so that the domain dimensions were $24\,h$ (length)$\times 12\,h$ (width)$\times 12\,h$ (height). Xie and Castro (2006) recommend a resolution of at least 20 grid cells per building height as sufficient for LES of staggered arrays of cubes. In the present case Castro et al. (2017) demonstrate that the present LES results for the mean flow and turbulence are close to the DNS results computed at higher resolution and lower Reynolds number.

The flow and turbulence in and above the building arrays were simulated as a fully-developed half-channel. The top boundary condition was a free-slip boundary that enforces zero shear stress at the top boundary of the domain. The lateral boundary conditions were periodic for the flow variables and scalar concentrations. Because the scalar concentration fields are spatially developing, the periodic conditions, which were used for the flow variables, cannot be employed at the outflow. In OpenFOAM the solver still formally used the periodic boundary conditions for scalars, but cut zones, in which the concentration fields were set to zero, were placed at the outlet of the domain. These zones serve as the outflow boundary condition. In ELMM the outflow boundary conditions for scalar concentration depend on the instantaneous flow direction. When the flow is into the domain at a given point of the boundary, the concentration outside of the domain is zero in the neighbouring cell. When the flow is out of the domain, the concentration gradient is set to zero.

#### Scalar Sources

All scalar sources used in the LES were localized ground-level sources with a scalar flux that was constant in time. The shape of the source was set to be close to circular used in the wind-tunnel experiments, but smaller due to the limitations of the grid with finite resolution. Twelve grid cells distributed around the centre of the source were used to represent the localized source. All cells containing the source had the same scalar flux. The area of the source in the finite grid is the same as the area of a circle with diameter $0.244\,h$, compared with $0.3\,h$ in the wind tunnel. The constant-flux boundary condition was implemented by injecting the appropriate amount of scalar into neighbouring fluid cells at each timestep.

The scalar sources were placed in three different positions with respect to the regular array building block: in the centre of the long street, in the centre of the short street and in the centre of the intersection of the streets. The source positions are numbered as shown in Fig. 1c. For the case with a tall building present the scalar sources were placed in similar locations around the building and the source numbers are presented in Fig. 1d. The wind-tunnel experiments and the source positions used are summarized in Table 1 while the configuration of the LES runs is presented in Table 2.

**Table 2:** Configurations of the LES simulations

LES model	Buildings	Domain size (*h*)	Wind dir. ($^{\circ }$)	Source positions
OpenFOAM & ELMM	Uniform	$12\times 12\times 12$	0	S1, S2, S3
OpenFOAM & ELMM	Uniform	$12\times 12\times 12$	45	S1, S2, S3
OpenFOAM & ELMM	Uniform	$12\times 12\times 12$	90	S1, S2, S3
OpenFOAM	Tall building	$24\times 12\times 12$	0	S1–S8
ELMM	Tall building	$24\times 12\times 12$	0	S1

Domain size in units of *h* are expressed as length $\times$ width $\times$ height

**Fig. 2:**
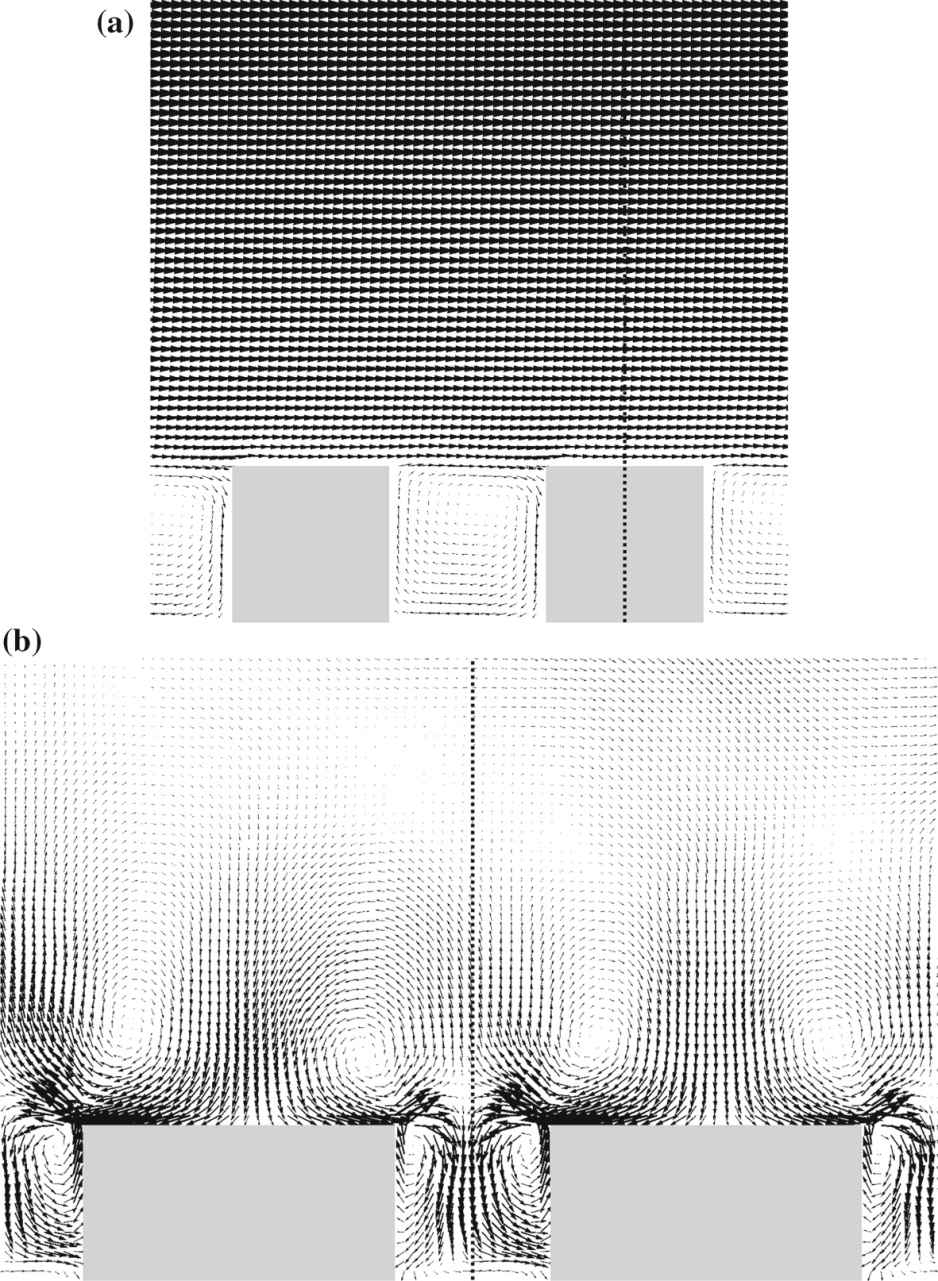
Mean flow vectors in the array of regular buildings at wind direction $0^{\circ }$ simulated by OpenFOAM: **a** the $x{-}z$ plane at $y = 0$, **b** the $y{-}z$ plane at $x = 0$ with vectors scaled $20\times$relative to **a**. The dotted line is the *z* axis

## Regular Array of Buildings

### Flow and Turbulence

A detailed analysis of the flow and turbulence in and above the regular array can be found in Castro et al. (2017). Here we summarize the results most relevant to the scalar dispersion in the same geometry.

The character of the flow within the canopy strongly depends on the wind direction. The planar area density of the building array has the same value for all wind directions, $\lambda _{\mathrm {p}}=0.33$; on the other hand the frontal area density $\lambda _{\mathrm {f}}$ depends on wind direction. For the $90^{\circ }$ wind direction the shorter face of the blocks faces into the flow (Fig. 1a) and $\lambda _{\mathrm {f}}$ has the smallest value of , while at the $0^{\circ }$ wind direction the blocks face the flow with their longest walls, implying a larger value of . An even larger value $\lambda _{\mathrm {f}}=0.35$ is encountered at the wind direction of $45^{\circ }$. Also, there are no continuous streets oriented parallel to the wind direction at $45^{\circ }$. These changes produce differences in the drag exerted by the canopy at different wind directions, where the highest drag is found for the $45^{\circ }$ case and the smallest for the $90^{\circ }$ wind direction.

The average flow inside the canopy strongly depends on the wind direction. For wind directions of $0^{\circ }$ and $90^{\circ }$ the area can be divided into street canyons that are perpendicular to the mean wind direction and dominated by a recirculating flow and street canyons parallel to the flow that form channels in which the flow is in a single direction over a long distance. In the intermediate $45^{\circ }$ case both the short streets and the long streets show spiralling flow, a combination of motion along the streets and rotating around the street axis. Especially in the long streets the flow is channelling and creates a classical street-canyon flow with a large flow component along the street axis. An example of the mean flow at wind direction $0^{\circ }$ is shown in Fig. 2. An important feature is the negative vertical velocity above the centre of the short streets, which influences the scalar fluxes.

### Mean Scalar Concentrations

To be able to compare simulations and measurements made at different scales, a dimensionless concentration is commonly used, defined as

5 $$\begin{aligned} c^{*}&= c\frac{U_{\mathrm {ref}}L_{\mathrm {ref}}^{2}}{Q}, \end{aligned}$$

where $c^{*}$ is the characteristic dimensionless concentration, *c* is the measured or computed concentration, $U_{\mathrm {ref}}$ is a characteristic flow speed, $L_{\mathrm {ref}}$ is a characteristic length and *Q* is the source rate. The characteristic length was set to $L_{\mathrm {ref}}=h$. Because the experimental and simulated wind profiles differ above $z/h\approx 3$ (Castro et al. 2017) the mean velocity over the array at $z=2.8\,h$ was chosen as $U_{\mathrm {ref}}$. Another reason for this choice is that the flow velocity at a certain height can be directly measured more easily in the wind tunnel or outdoors than can the friction velocity.

The results available from the FFID and simultaneous LDA measurements are point-wise values of the mean concentration *C*, the concentration variance $\overline{c^{\prime 2}}$, horizontal turbulent scalar fluxes $\overline{u^{\prime }c^{\prime }}$ and $\overline{v^{\prime }c^{\prime }}$ and the horizontal advective fluxes *UC* and *VC*.

One obvious feature of the measured mean concentration fields, which is not present in the LES results, is the strong asymmetry of the plume at the $0^{\circ }$ wind direction. Because the boundary conditions are supposed to be symmetrical with regards to the $x{-}z$ plane, one would expect symmetrical results along this plane. It is very difficult if not impossible to achieve perfect symmetry in the wind tunnel.

However, the experimental methods have been improved since those reported in Castro et al. (2017). The accuracy of the turntable rotation, controlling the orientation of the model in the wind tunnel, was about $0.5^{\circ }$. However, final alignment was achieved manually, reducing the alignment error to $0.25^{\circ }$ (actually measured as a displacement around the circumference of the turntable). Uncertainty in array element locations was typically up to about $1\,\mathrm {mm}$.

Data collection times were selected to control the standard error in the results, leading to a typical standard error in *U* and *C* of $2\%$, in $\overline{u^{\prime 2}}$ of $10\%$, and in $\overline{v^{\prime 2}}$ and $\overline{w^{\prime 2}}$ of $5\%$. The exact $0^{\circ }$ wind direction is a special case because of possible symmetry breaking (see later). It is unlikely to be present in the real atmosphere and the applicability of the results for this case to real situations is uncertain.

**Fig. 3:**
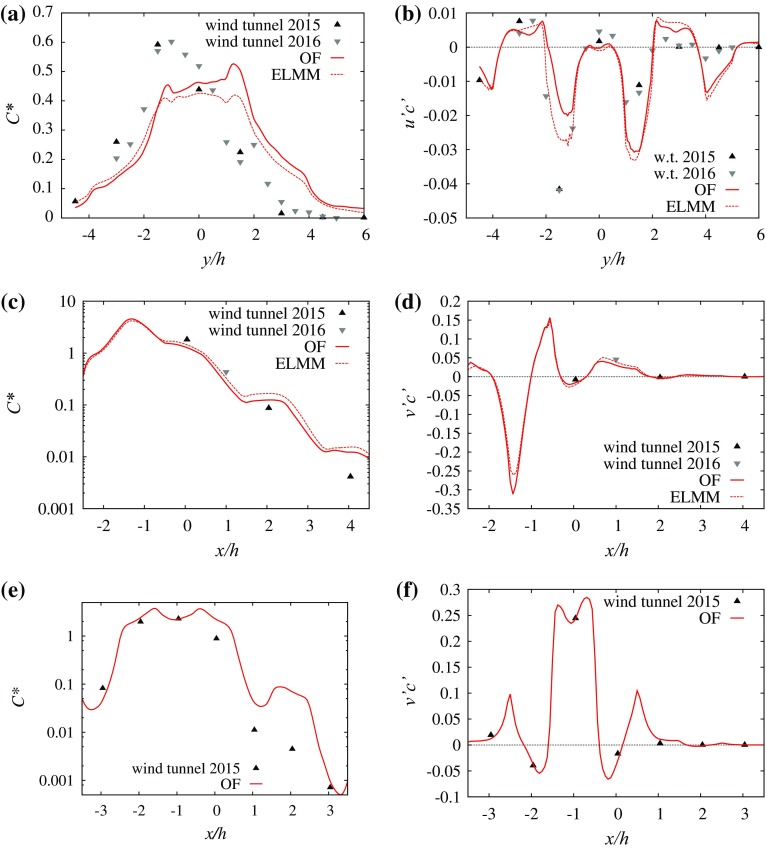
Selected profiles of measured and simulated mean concentrations and turbulent scalar fluxes from source S1. **a** $C^{*}$at $x=1\,h$, $z=0.5\,h$ and $0^{\circ }$ wind direction, **b** $\overline{c^{*\prime }u^{\prime }}$at $x=1\,h$, $z=0.5\,h$ and $0^{\circ }$ wind direction, **c** $C^{*}$at $y=-1.5\,h$, $z=0.5\,h$ and $45^{\circ }$ wind direction, **d** $\overline{c^{*\prime }v^{\prime }}$at $y=-1.5\,h$, $z=0.5\,h$ and $45^{\circ }$ wind direction, **e** $C^{*}$at $y=-1.5\,h$, $z=0.5\,h$ and $90^{\circ }$ wind direction, **f** $\overline{c^{*\prime }v^{\prime }}$at $y=-1.5\,h$, $z=0.5\,h$ and $90^{\circ }$ wind direction

The measured scalar concentrations from source S1 (the long street centre) are plotted in Fig. 3a. It appears that in the present case there is a tendency for a spanwise component of the flow in the canopy in the wind-tunnel experiment. This feature is pronounced especially for source S1 that is supposed to lie exactly on the dividing streamline, where the mean flow is diverging either to the left or to the right. The measurement results show preferential transport in the negative *y* direction and channelling into the neighbouring short street.

At the $45^{\circ }$ wind direction the centreline of the plume at $z/h=0.5$ is not aligned with the mean wind vector above the canopy anywhere in the computational domain. The channelling effect is stronger for the long streets than for the short streets. This difference is more evident for the dispersion from the scalar source located in the centre of the long street (Fig. 3c, d). Above the canopy the transport is dominated by advection by the mean flow in a direction close to $45^{\circ }$.

The $90^{\circ }$case in Fig. 3e is again asymmetric, for this case along the $y{-}z$ plane. An important feature of the dispersion from source S1 at the $90^{\circ }$ wind direction is the large difference between the measured mean concentrations at $z/h=0.5$ and $z/h=1.5$ at the plume centreline. At position $x/h=-0.5$, $y/h=-4.5$ (downwind distance $3\,h$ from the source) the ratio of the dimensionless mean concentrations $C_{z=0.5h}^{*}/C_{z=1.5h}^{*}=29.9$, indicating that vertical scalar dispersion is small in this configuration. In a comparable situation at $0^{\circ }$ wind direction and with dispersion from source S3 the ratio is only $C_{z=0.5h}^{*}/C_{z=1.5h}^{*}=5.28$.

**Fig. 4:**
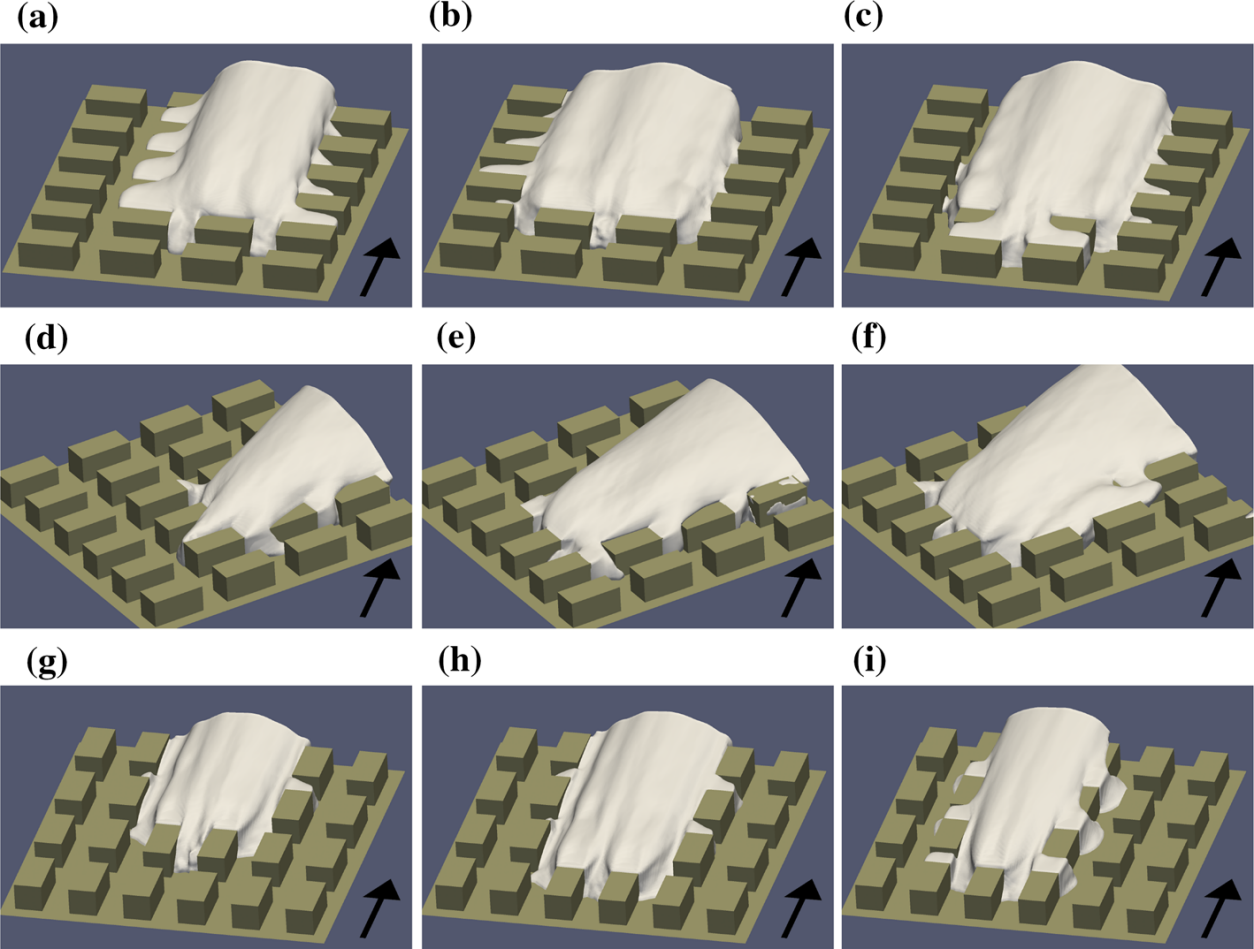
The isocontours of mean concentration $C^{*}=0.1$. Wind directions and source positions: **a** $0^{\circ }$, S1; **b** $0^{\circ }$, S2; **c** $0^{\circ }$, S3; **d** $45^{\circ }$, S1; **e** $45^{\circ }$, S2; **f** $45^{\circ }$, S3; **g** $90^{\circ }$, S1; **h** $90^{\circ }$, S2; **i** $90^{\circ }$, S3. The black arrows denote the wind direction. (OpenFOAM)

Surfaces of constant numerically-simulated mean concentration $C^{*}=0.1$ are plotted in Fig. 4. One can immediately note the difference in the mean plume direction at $45^{\circ }$ wind direction and the difference in the plume width between different configurations.

### Wind Direction Sensitivity

In LES the same three wind directions were examined, but in addition to the source position used in the experiments (long street centre), the sources positioned in the short street centre ($x/h=0$, $y/h=0$) and the centre of the intersection ($x/h=-0.5$, $y/h=0$) were considered.

The first step of the analysis was validation of the model predictions against the available experimental data. The validation of the flow results was already performed in Castro et al. (2017). One can notice the difference in the plume shape for a wind direction of $0^{\circ }$ and a scalar source position in the long street centre in Fig. 3a. For both LES models the simulated plume is essentially symmetric after sufficient averaging time (more than $750\,h/u_{\tau }$ was used).

**Fig. 5:**
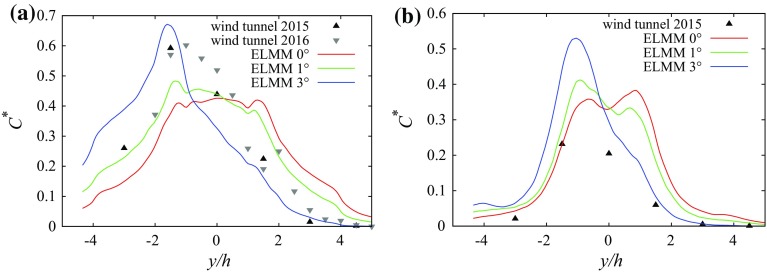
Sensitivity of mean dimensionless concentration from source S1 to small changes in wind direction: **a** along line $x/h=1,\,z/h=0.5$, **b** along line $x/h=1,\,z/h=1.5$

To investigate the sensitivity of the $0^{\circ }$ configuration further sensitivity tests were performed using ELMM, where the wind direction was shifted by $1^{\circ }$ and by $3^{\circ }$ from the base configuration. The comparison of spanwise profiles of mean concentrations is shown in Fig. 5. It is apparent that the experimental results are closer to the shifted profiles and correspond to a wind direction between $1^{\circ }$ and by $3^{\circ }$. This does not necessarily mean that the experimental model was set at an (incorrect) angle of this magnitude. Other effects such as possible spanwise structures in the flow in the wind tunnel, inexact building alignment or a small difference in the source position could produce similar results. Additionally, simulation results of the same problem on a smaller domain ($6\,h\times 6\,h\times 6\,h$) showed a significant spanwise velocity component even in the time-averaged flow. This was consistent for different models and grid resolutions, suggesting that the flow is susceptible to symmetry breaking.

The plume shape strongly depends on the source position (Fig. 4). In the $0^{\circ }$ case a considerable difference in plume behaviour is found between the source position in the centre of the street canyon of the long street and the other two source positions. When the source is located in a street oriented perpendicular to the wind direction, the scalar is first well mixed within the street canyon and then released into the shear layers at the top and at the sides of the street canyon.

### Scalar Fluxes

For parametrizations of street-network dispersion models the most important quantities are the scalar fluxes at the canopy top and at the boundaries of individual streets and intersections. The total scalar flux $\psi _{\mathrm {tot}}$ can be divided into two components, the advective scalar flux $\psi _{\mathrm {adv}}$ and the turbulent scalar flux $\psi _{\mathrm {turb}}$ defined as

6 $$\begin{aligned} \psi _{\mathrm {tot}}^{u}&= \overline{cu}=CU+\overline{c^{\prime }u^{\prime }}, \end{aligned}$$

7 $$\begin{aligned} \psi _{\mathrm {adv}}^{u}&= CU, \end{aligned}$$

8 $$\begin{aligned} \psi _{\mathrm {turb}}^{u}&= \overline{c^{\prime }u^{\prime }}, \end{aligned}$$

where the velocity component *u* determines the streamwise component of the flux.

The dimensionless scalar flux is computed as

9 $$\begin{aligned} \psi ^{*}&=\psi \frac{L_{\mathrm {ref}}^{2}}{Q}=\psi \frac{h^{2}}{Q}, \end{aligned}$$

and in LES it is possible to deduce both flux components explicitly. The scalar fluxes control the dispersion and determine the shape of the plume. The most interesting set of scalar fluxes are the vertical fluxes at the roof-top level, which control the exchange of the scalar between the canopy and the boundary layer above. The scalar flux components,

10 $$\begin{aligned} \psi _{\mathrm {adv}}^{w*}&=CW\frac{h^{2}}{Q}, \end{aligned}$$

and

11 $$\begin{aligned} \psi _{\mathrm {turb}}^{w*}&=\overline{c^{\prime }w^{\prime }}\frac{h^{2}}{Q} \end{aligned}$$

clearly depend on the mean vertical velocity *W* and the turbulent fluctuations $w^{\prime }$. In regions where the flow is parallel to the street the mean vertical velocity component is small and we can expect a relatively small magnitude of the vertical advective scalar flux and a large contribution of the turbulent flux to the total flux.

We define the integrated vertical scalar flux as

12 $$\begin{aligned} \Psi ^{a,b}&=\iint _{x/h\in (a,\,b),\;y/h\in (-L_y/2,\,L_y/2),\;z/h=1}\psi ^{w*}\mathrm {\,d}\left( \frac{x}{h}\right) \mathrm {\,d}\left( \frac{y}{h}\right) \end{aligned}$$

where $L_y$ denotes the size of the domain in the *y* direction. The integration is performed on the surface of $z/h=1$ in strips between two constant values (*a* and *b*) of *x* across the entire span of the domain in the *y* direction. Profiles of vertical scalar fluxes at the centreline of the top of several streets show that the turbulent flux component generally has a larger magnitude than the advective flux component. This is due to the uniform height and flat roofs that allow flow to be almost parallel to the roofs over the canopy.

**Fig. 6:**
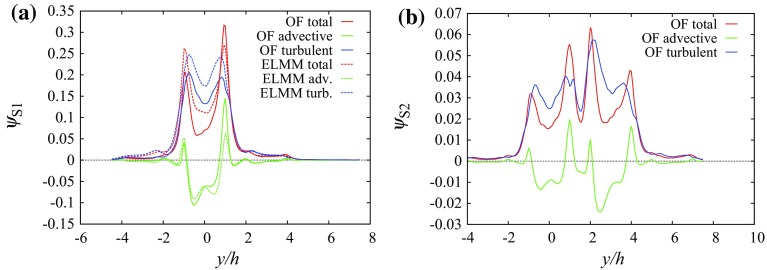
Vertical scalar fluxes along line $x/h=-1,\,z/h=1$ (the centreline of the canyon containing source S1) at the $0^{\circ }$ wind direction: **a** source S1, **b** source S2

At the $0^{\circ }$ wind direction Fig. 6 shows the vertical scalar fluxes having a mostly negative advective flux above the centre of street $x/h=-1$ with several positive peaks for sources S1 and S2. The total scalar flux is dominated by the turbulent flux component. The large positive vertical scalar flux from source S1 above the source street produces transport of a significant part of the scalar above the canopy.

The integrated total flux through the top boundary between $x=-1.5$ and $x=-0.5$ (a strip which includes the street in which source S1 is located) is

13 $$\begin{aligned} \Psi _{\mathrm {tot,}S1}^{-1.5,-0.5}&\doteq 0.51. \end{aligned}$$

Approximately one half of the scalar released from source S1 is transported to the external flow above the street containing source S1. The concentrations above the next long street canyon at $x/h=1$ behind the building $y\in [-1.5,1.5]$ are larger than the concentrations inside the canyon. This causes the scalar fluxes to be negative in that area and in the subsequent canyons.

**Fig. 7:**
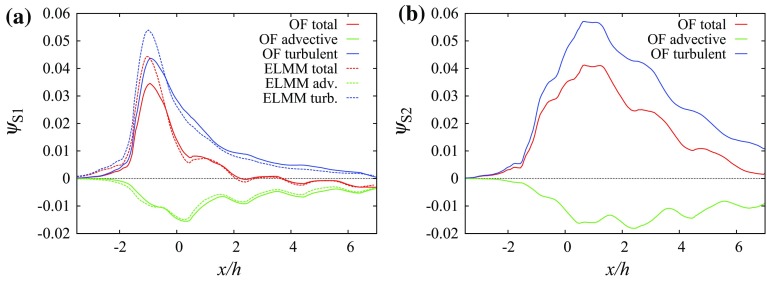
Vertical scalar fluxes along line $y/h=1.5,\,z/h=1$ (the centreline of the short streets containing sources S2 and S3) at the $0^{\circ }$ wind direction: **a** source S1, **b** source S2

Over the street centreline at $y/h=1.5$, Fig. 7 shows that the advective flux component is negative for both S1 and S2 due to the downward mean vertical flow shown in Fig. 2b. For S1 the total flux is positive due to the turbulent flux up to $x/h\approx 2$ and values close to zero above that height, while for S2 it remains positive in the simulated domain.

**Fig. 8:**
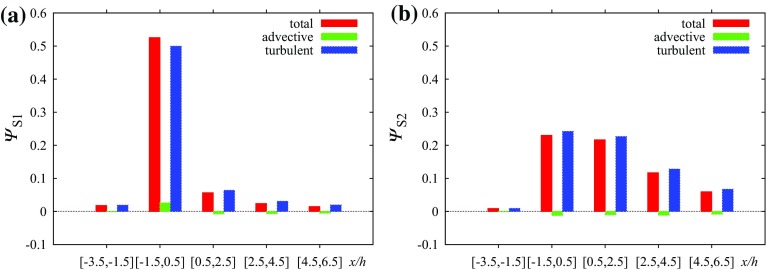
Scalar fluxes in the uniform height array on surface $z/h=1$ integrated over strips oriented in the *y* direction and width 2*h* in the *x* direction: **a** source S1, **b** source S2

The fluxes integrated in strips 2*h* wide in Fig. 8 show that the contribution of advective fluxes to the total flux is small. It is also clear that a larger part of the flux is released upwards from the source canyon for source S1 while S2 is released over a larger distance from the source. The value of $\varPsi _{\mathrm {tot,}S1}^{-3.5,6.5}$ is 0.63 and $\varPsi _{\mathrm {tot,}S2}^{-3.5,6.5}\doteq 0.67$, implying that more scalar is being released above the canopy for source S2 even though for source S1 a large part is released immediately above the street containing the source.

## Tall Building in the Array of Regular Buildings

### Flow and Turbulence

**Fig. 9:**
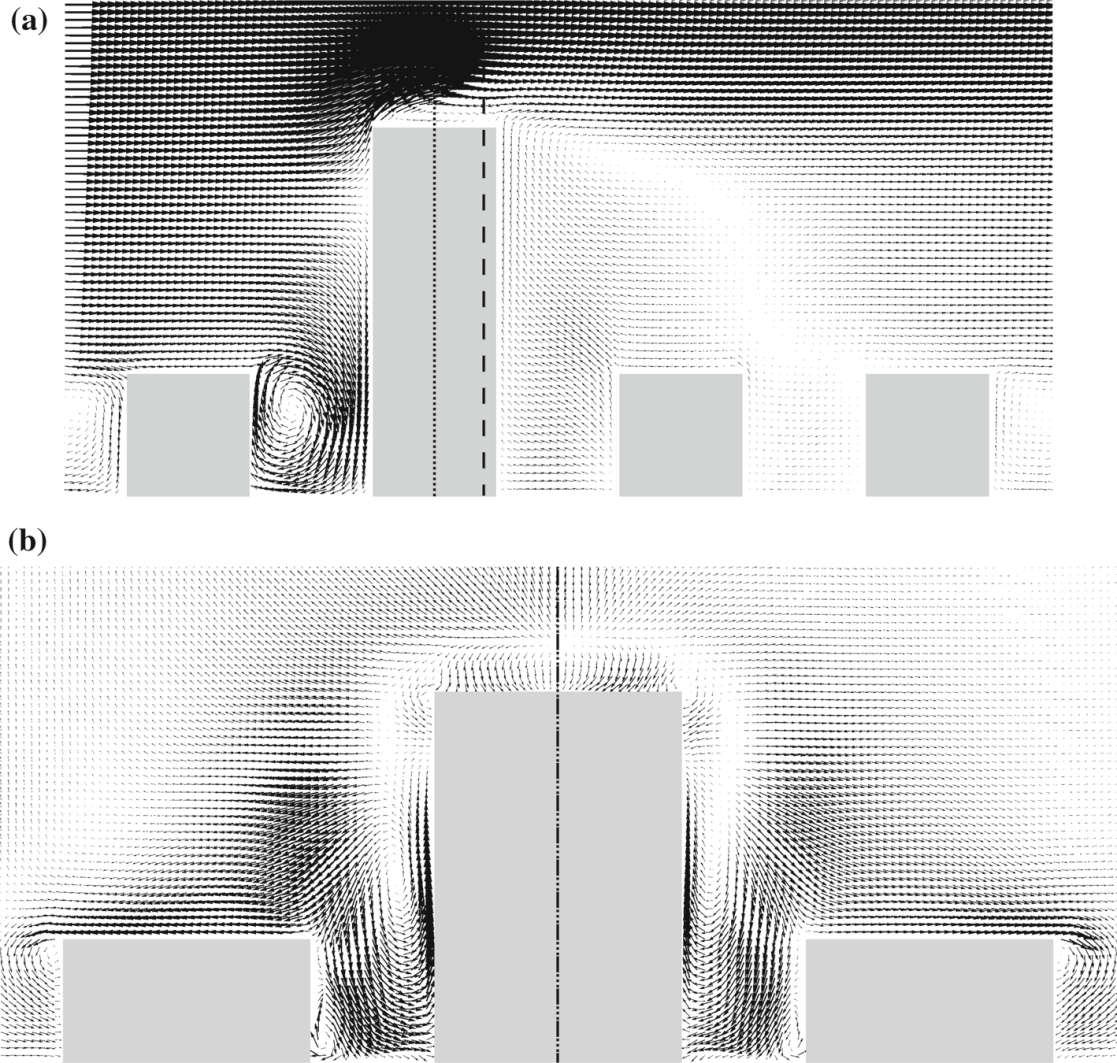
Mean flow vectors near the tall building at the $0^{\circ }$ wind direction simulated by OpenFOAM: **a** the $x{-}z$ plane at the centre of the tall building. **b** The $y{-}z$ plane at $x/h=0.4$ with vectors scaled $4\times$ relative to **a**. The dotted line in (**a**) is the *z*-axis $(x = 0)$ and the dashed line denotes the *x*-location of the *y*–*z* plane shown in (**b**). In (**b**) the dash-dot line denotes the $y = 0$ plane

**Fig. 10:**
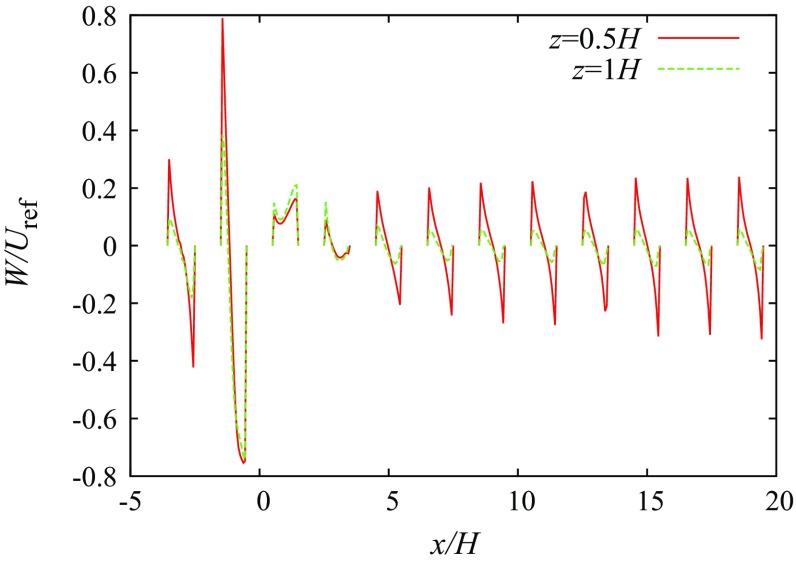
The vertical velocity along two lines at $y/h=0$ and two vertical levels (OpenFOAM)

A tall building in the regular array constitutes an additional obstacle to the flow. The flow pattern around the part of the building that surmounts the regular buildings is similar to the flow around an isolated building. Only the $0^{\circ }$ wind direction is considered here, with the flow visualized as in Fig. 9. In the side view in Fig. 9a one can notice the downdraft at the front of the building and the wake behind it. The downdraft and the wake also strongly affect the recirculation in the street canyons in front of and behind the building. While in front of the building the recirculation is strongly increased, there is no recirculation apparent in the first canyon behind the building and the recirculation in the second canyon is strongly reduced as documented in Fig. 10, which shows the vertical motion at two vertical levels. The downdraft also causes the vertical motion at the roof top of the front canyon to be mainly downward, which is compensated by horizontal flow outward from the canyon at its ends.

In the $y{-}z$ plane shown in Fig. 9b one can note the strong downward flow in and above the street canyon. The tall building also produces a noticeable spanwise flow component above the roofs of the regular buildings on the left and on the right of the tall building pointing away from the tall building. The mean wind direction at a height $0.1\,h$ above the roof of the left and right neighbouring buildings varies between $10^{\circ }$ and $20^{\circ }$ outwards.

**Fig. 11:**
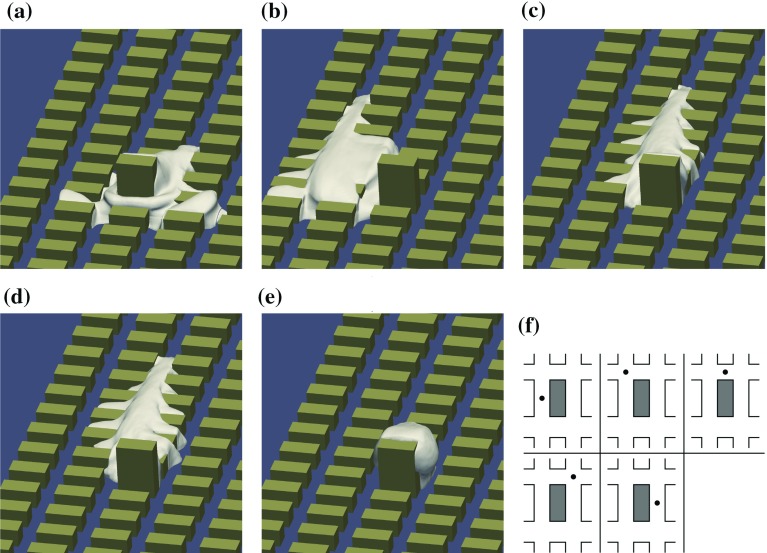
The isocontours of mean concentration $C^{*}=0.1$ at the $0^{\circ }$ wind direction. Periodic boundary conditions are applied and two copies of the concentration fields are depicted with one copy translated in the *y* direction. Sources: **a** S1, **b** S2, **c** S3, **d** S4 and **e** S5. **f** the source location relative to the tall building. (OpenFOAM)

### Scalar Fluxes Near the Tall Building

There were eight point scalar sources placed at distinct locations on the ground close to the tall building in the simulation. These locations correspond to the locations used in the regular array, i.e. the long street centre, the short street centre and the intersection centre (see Fig. 1c). Due to the symmetry of the problem around the $x{-}z$ plane crossing the tall building centre only five of the locations are unique (Fig. 11). In this way more accurate results can be achieved by using the ensemble average of each two symmetric cases. Therefore the reported OpenFOAM results for sources S2, S3, and S4 are actually ensemble averages of results from source S2 and results from source S8 mirrored around the $x{-}z$ plane, from source S3 and from source S7 mirrored around the $x{-}z$ plane, and from source S4 and from source S6 mirrored around the $x{-}z$ plane, respectively. In ELMM only one source in front of the building was simulated.

Figure 11 shows isocontours of mean concentration for the five distinct source positions; clearly, the presence of the tall building strongly influences the shape and width of the plume. In addition to the well-known phenomenon of flow and dispersion around an isolated building, the interaction with the regular building array is important.

The front side downdraft and the strong recirculation in the front canyon causes the scalar from the canyon centre source to mainly spread sideways. The isocontours for source S1 in Fig. 11a form a horseshoe-like structure around the tall building. In this case the plume becomes wider than the domain width ($12\,h$) for a small enough concentration threshold and the influence of the periodic lateral boundary conditions is apparent in Fig. 11a.

The negative vertical velocity at the centre of the front canyon at the rooftop level leads to large negative advective scalar flux for the ground source at the centre of the canyon. The turbulent scalar flux has the opposite sign, due to the sign of the mean concentration gradient, but the magnitude is much smaller. However, in addition to the main peak of the turbulent scalar flux in the main canyon there is a local minimum (negative) at the end of the canyon and a local maximum (positive) of the turbulent scalar flux in the intersections on both sides of the front canyon. These are connected to the local structure in the vorticity field above the intersection attached to the regular building in front of the tall building (Fig. 13a). The structure is analogous to the horseshoe vortex of an isolated building. The effective turbulent diffusivity above the intersection is negative (Fig. 13b) while the mean concentration vertical gradient is positive (not shown). This is also the location where the horseshoe-like structure in the mean concentration field in Fig. 11a crosses the roof level.

An analysis of time series of *w* and *c* from source S1 in the centre of the intersection $(x/h,\,y/h,\,z/h)=(-1,\,-1.5,\,1)$ has shown that the concentration is intermittent there. Only for 60% of the time is the concentration larger than 1% of its mean value. Quadrant analysis has revealed that $c^{\prime }>0$ for 23% of the time and the largest part of the turbulent flux occurred in the first quadrant ($w^{\prime }>0$, $c^{\prime }>0$). That means that, although the mean concentration increases with height at this location, the dominant vertical scalar flux arises intermittently from the region of the intersection below $z/h=1$.

**Fig. 12:**
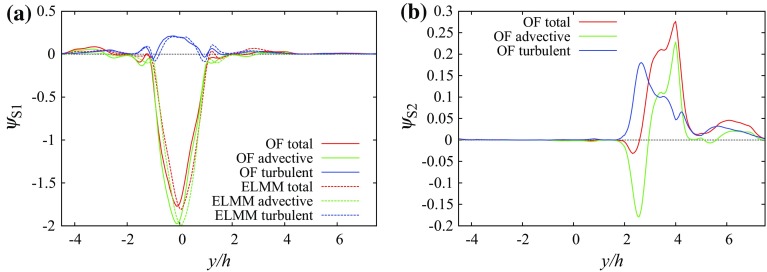
Vertical scalar fluxes along line $x/h=-1,\,z/h=1$ (the centreline of the canyon in front of the tall building at roof level): **a** source S1, **b** source S2

**Fig. 13:**
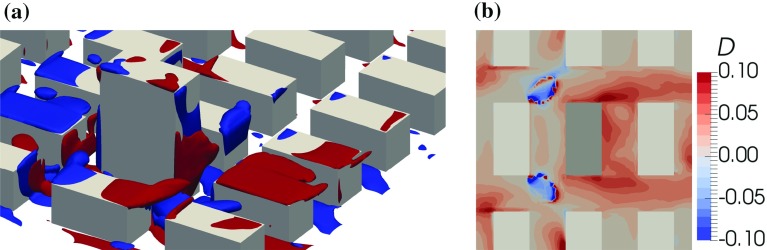
Vorticity and turbulent diffusivity in front of the tall building (OpenFOAM): **a** isocontours of vorticity component $\omega _{x}=-U_{\mathrm {ref}}/h$ (blue) and $\omega _{x}=U_{\mathrm {ref}}/h$ (red), **b** turbulent diffusivity for scalar from source S1 in vertical direction defined as $D=-\psi _{\mathrm {turb,}S1}^{w*}/\left( \frac{\partial C_{S1}^{*}}{\partial (z/h)}\right)$ at $z/h=1$

For source S2 the vertical fluxes sampled along line $x/h=-1,\,z/h=1$ are very different as seen in Fig. 12a. The strong flow pointing away from the tall building in the *y*-direction causes horizontal flow towards the street canyon left of the source (Fig. 11b). The canyon acts as a secondary source. Above the intersection ($y/h\in (1.5,\,2.5$)) the vertical velocity component and the advective scalar flux are negative while the turbulent flux is positive with similar magnitude. Above the street canyon both fluxes are positive, leading to a larger value of the total scalar flux (Fig. 13).

**Fig. 14:**
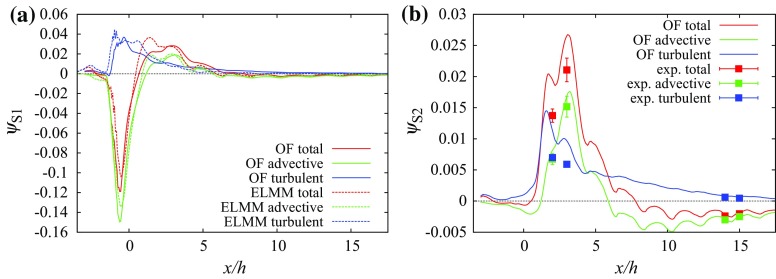
Vertical scalar fluxes along line $y/h=1.5,\,z/h=1$ (the centreline of the short streets left of the tall building at roof level): **a** source S1, **b** source S2. The error bars in **b** correspond to the standard deviation of the measured values. Other sources of uncertainty (e.g., due to positioning of the velocity and the scalar probe in areas with large scalar gradients) cannot be excluded

When comparing the scalar fluxes from the same two sources along line $y/h=1.5,\,z/h=1$ we can identify a large difference between the two sources at $x/h\in (\approx 0,\,\approx 2$) (Fig. 14). The strong downward motion in the short street (cf. Fig. 9b) produces a negative peak in the advective scalar flux for source S1, and connected with a negative peak for the total scalar flux because the positive turbulent flux has a smaller magnitude. This is possible because the scalar is already mixed to a certain degree with non-zero concentrations above the roof level. That is not true for the scalar from source S2, which has small concentrations in this interval and all scalar fluxes are small. In the interval $x/h\in (\approx 0.5,\,\approx 5.5$) the turbulent and advective scalar fluxes are positive for both sources due to the positive vertical velocity in the recirculation zone behind the building, but after $x/h\approx 5.5$ the advective fluxes become negative again. For both scalars from sources S1 and S2 the magnitude of the advective vertical flux becomes larger than that of the turbulent flux between $x/h=7.7$ and $x/h=7.8$ and the turbulent flux magnitude decays faster with distance from the source than does the advective flux magnitude. As a result, the total flux magnitude does not decay much after $x/h=10$ and the flux remains negative on the selected line $y/h=1.5,\,z/h=1$.

**Fig. 15:**
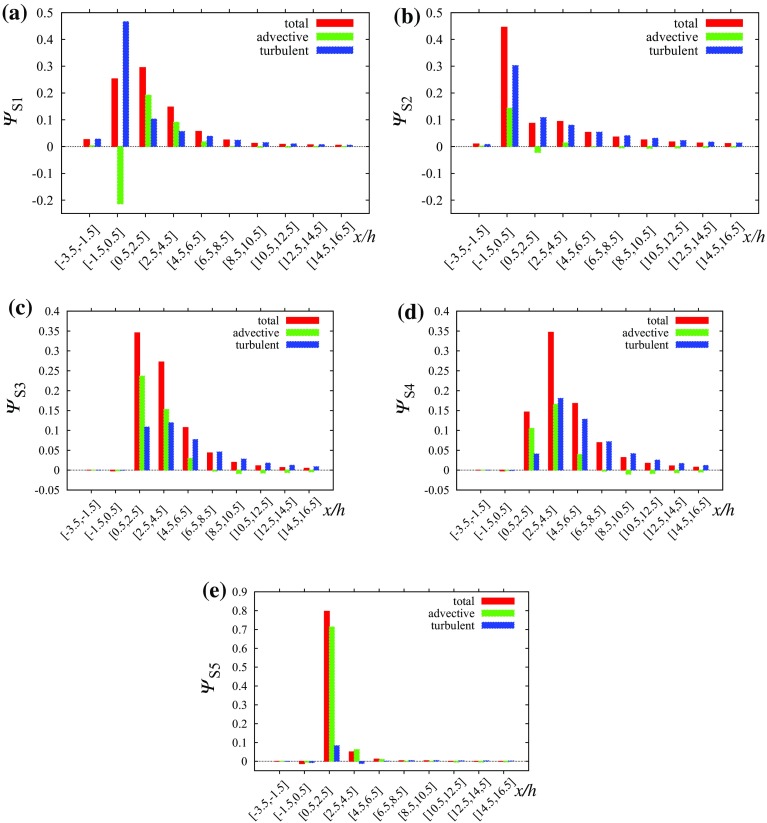
Scalar fluxes in the array containing the tall building on surface $z/h=1$ integrated over strips oriented in the *y* direction and of width 2*h* in the *x* direction: **a** source S1, **b** source S2, **c** source S3, **d** source S4 and **e** source S5

However, the street centreline fluxes are affected by the negative vertical flow velocities also existing in the uniform height array (Fig. 2b); in other locations in the street network the vertical velocities are different. The fluxes integrated over the roof-top surface ($z/h=1$) in Fig. 15 represent the true contribution of each flux component to the total scalar exchange. For source S1 the transport of the scalar above the canopy is delayed in comparison with the uniform height array and the maximum of the total flux is located in the interval behind the tall building. The large turbulent flux at the top of the long street containing the source is balanced by a large negative advective flux. In the first two strips behind the tall building the advective flux is positive and larger than the turbulent flux due to the positive vertical velocity in the building wake.

For source S2 the advective flux in the source’s long street is positive. The total flux at $x\in [-1.5,0.5]$ is larger than for source S1 and also larger than in the uniform height array. Because the plume is shifted laterally away from the tall building (Fig. 11b), the positive vertical velocities in the wake influence the fluxes from source S2 less than from S1. There is still a secondary maximum in the total flux at $x\in [2.5,4.5]$ due to a positive advective flux for source S2.

The value of $\Psi _{\mathrm {tot,}S1}^{-3.5,16.5}$ is 0.76 while $\Psi _{\mathrm {tot,}S2}^{-3.5,16.5}$ is equal to 0.68. That means that more scalar is being released above the canopy for source S1. Also, $\Psi _{\mathrm {tot,}S2}^{-3.5,6.5}\doteq 0.55$ while in the regular array case the value was 0.67. That means the vertical transport of the pollutant is weaker in the tall building scenario for source S2. For source S1 the vertical transport is enhanced by the tall building, $\Psi _{\mathrm {tot,}S1}^{-3.5,6.5}\doteq 0.69$ in comparison with the regular array value 0.63.

For sources S3 and S4 the situation is similar to source S2. They are also influenced by the negative vertical wind velocities on the sides of the tall building. Scalar from source 2 is partially transported horizontally behind the building where it is lifted in the wake by the positive vertical wind velocities above the canopy. This causes the peak of the advective flux at $x\in [0.5,2.5]$ (Fig. 15c). For source S2 this effect is reduced and the largest advective flux happens in the next street canyon in strip $x\in [2.5,4.5]$ (Fig. 15d). For both sources S3 and S4 the turbulent flux becomes dominant in strip $x\in [4.5,6.5]$ and beyond in the *x* direction similarly to source S2.

Source S5 lies directly in the wake of the building and the positive vertical velocities there control dispersion from this source (Fig. 15e). The total vertical scalar flux (dominated by the advective component) is approximately 0.80 in strip $x\in [0.5,2.5]$ and 0.05 in $x\in [2.5,4.5]$ and the majority of the scalar is elevated above the roof level in this area.

## Conclusions

Turbulent flow and scalar dispersion in an array of uniform-height buildings (regular array) were compared with flow and dispersion in an array with one building three times taller. Scalar dispersion in the uniform-height array showed strong sensitivity to small changes in the wind direction when the approaching flow faced the longest face of the building. This implied that great care needs to be taken in arranging the wind-tunnel model so as to produce a symmetric flow field. It also implies that long time averaging is necessary in LES to converge to nearly symmetric mean fields.

The finite spanwise dimension of the computational domain and the periodic lateral boundary conditions imply that care must be taken when interpreting the simulation results. As mentioned in Sect. 2.3.2 the geometry is actually a part of a large array with periodically repeated tall buildings. The recycling of the scalar plume through the lateral boundaries can happen in the simulation for locations with *x* larger than a certain value. Beyond this limit, the local values of concentrations and scalar fluxes will be larger than in a larger domain with repeated tall buildings. Thanks to the linearity of the scalar transport (Eq. 3) the fluxes integrated across the span of the domain are not affected.

Vertical scalar fluxes at the roof height in the regular array were dominated by the turbulent flux component for all three wind directions examined. The advective flux component was often negative but the larger positive turbulent flux determined the resulting positive sign of the total flux.

Depending on the position of the source in the array the bulk of the scalar plume can be below or above the roof level. If the source is placed in a recirculation zone in a street canyon the scalar is effectively transported upwards, similar to the results found in Brixey et al. (2009). If the source is in a street parallel to the wind direction the channelling in the street causes the scalar to be mainly advected horizontally and the vertical fluxes above the source street are small. This effect is enhanced by the negative vertical velocities found above those streets as described in Castro et al. (2017).

When a tall building is placed into the regular array the flow changes significantly, and larger vertical velocities allow significant advective vertical scalar fluxes. Scalar from ground-level sources in front of the tall building is mainly transported sideways around the building. Horizontal divergence of the flow is accompanied by downward vertical flow in front of the building that prevents the scalar reaching the top of the building at its windward side. The large recirculation zone behind the building causes upward transport of the scalar at the leeward side of the building. Due to the horizontal convergence of the flow, the plumes from sources located behind the tall building are narrower than those from sources located in front of the building (Fig. 11).

Integration of the vertical scalar fluxes over a large portion of the computation domain shows that the tall building can cause either an increase or a decrease of the vertical transport of the passive scalar from a localized source, depending on the source position relative to the tall building. A region with negative effective diffusion coefficient for vertical turbulent flux for a source in the centre of the street in front of the tall building has been found and connected with an intermittent flux of high concentration from below, against the mean concentration gradient in that position. Such regions are very difficult to simulate by simpler methods such as the Reynolds-averaged Navier-Stokes approach. The computed fields of scalar fluxes will be used to develop and improve parametrizations that approximate scalar fluxes in intersections and at the top boundaries of individual streets.

## Footnotes

1
http://www.diplos.org.
